# Pyrosequencing Revealed SAR116 Clade as Dominant *dddP*-Containing Bacteria in Oligotrophic NW Pacific Ocean

**DOI:** 10.1371/journal.pone.0116271

**Published:** 2015-01-23

**Authors:** Dong Han Choi, Ki-Tae Park, Sung Min An, Kitack Lee, Jang-Cheon Cho, Jung-Hyun Lee, Dongseon Kim, Dongchull Jeon, Jae Hoon Noh

**Affiliations:** 1 Marine Biotechnology Research Division, Korea Institute of Ocean Science and Technology, Ansan, Republic of Korea; 2 Department of Marine Biotechnology, Korea University of Science and Technology, Daejeon, Republic of Korea; 3 School of Environmental Science and Engineering, Pohang University of Science and Technology, Pohang, Korea; 4 Marine Ecosystem Research Division, Korea Institute of Ocean Science and Technology, Ansan, Republic of Korea; 5 Division of Biology and Ocean Sciences, Inha University, Incheon, Republic of Korea; 6 Ocean Circulation and Climate Research Division, Korea Institute of Ocean Science and Technology, Ansan, Republic of Korea; 7 Department of Marine Biology, Korea University of Science and Technology, Daejeon, Republic of Korea; CAS, CHINA

## Abstract

Dimethyl sulfide (DMS) is a climatically active gas released into the atmosphere from oceans. It is produced mainly by bacterial enzymatic cleavage of dimethylsulfoniopropionate (DMSP), and six DMSP lyases have been identified to date. To determine the biogeographical distribution of bacteria relevant to DMS production, we investigated the diversity of dddP—the most abundant DMS-producing gene—in the northwestern Pacific Ocean using newly developed primers and the pyrosequencing method. Consistent with previous studies, the major dddP-containing bacteria in coastal areas were those belonging to the Roseobacter clade. However, genotypes closely related to the SAR116 group were found to represent a large portion of dddP-containing bacteria in the surface waters of the oligotrophic ocean. The addition of DMSP to a culture of the SAR116 strain Candidatus Puniceispirillum marinum IMCC1322 resulted in the production of DMS and upregulated expression of the dddP gene. Considering the large area of oligotrophic water and the wide distribution of the SAR116 group in oceans worldwide, we propose that these bacteria may play an important role in oceanic DMS production and biogeochemical sulfur cycles, especially via bacteria-mediated DMSP degradation.

## Introduction

Dimethylsulfoniopropionate (DMSP) is produced mainly by phytoplankton and macroalgae in the ocean [1,2], and may function as an osmolyte, antioxidant, predator deterrent, and cryoprotectant [3–7]. DMSP released into seawater by cell breakage processes, such as predation [5] and viral lysis [8], is catabolized via two enzymatic pathways: the demethylation and cleavage pathways [9,10]. The demethylation pathway is a major DMSP catabolic pathway and a source of reduced sulfur and carbon for microbial cells [11,12]. Alternatively, the cleavage pathway is mediated via various DMSP lyases [13] and produces dimethyl sulfide (DMS) gas, which can be released from oceans and photochemically oxidized, acting as a cloud condensing nucleus [14]. Although only a minor proportion (2–21%) of dissolved DMSP is cleaved into DMS [15], a great deal of attention has been paid to the cleavage pathway because of the relationship between DMS and climate change [14].

To date, six DMSP lyases have been identified from bacterial isolates: DddY from *Alcaligenes faecalis* [16], DddD from *Marinomonas* sp. and *Ruegeria pomeroyi* [10], DddL from *Sulfitobacter* sp. and *Rhodobacter sphaeroides* [17], DddP from *Roseovarius nubinhibens* and *Ru. pomeroyi* [18], DddQ from *Ro. nubinhibens* and *Ru. pomeroyi* [19], and DddW from *Ru. pomeroyi* [20]. Most lyases (DddY, DddP, DddQ, DddL, and DddW) cleave DMSP into DMS and acrylate, but DddD generates DMS and 3-hydroxypropionate from DMSP. Among the six DMSP lyases, *dddP* and *dddQ* genes were found to be most abundant in the Global Ocean Sampling (GOS) data set, indicating that they play important roles in ocean DMS production [19]. However, studies on the diversity and biogeography of these genes are rare [21,22].

To identify bacterial diversity related to DMS production, we established a transect from coast to tropical open ocean in the northwestern (NW) Pacific Ocean, and studied distribution of *dddP* gene diversity using a newly designed primer pair and amplicon pyrosequencing method.

## Materials and Methods

**Figure 1:**
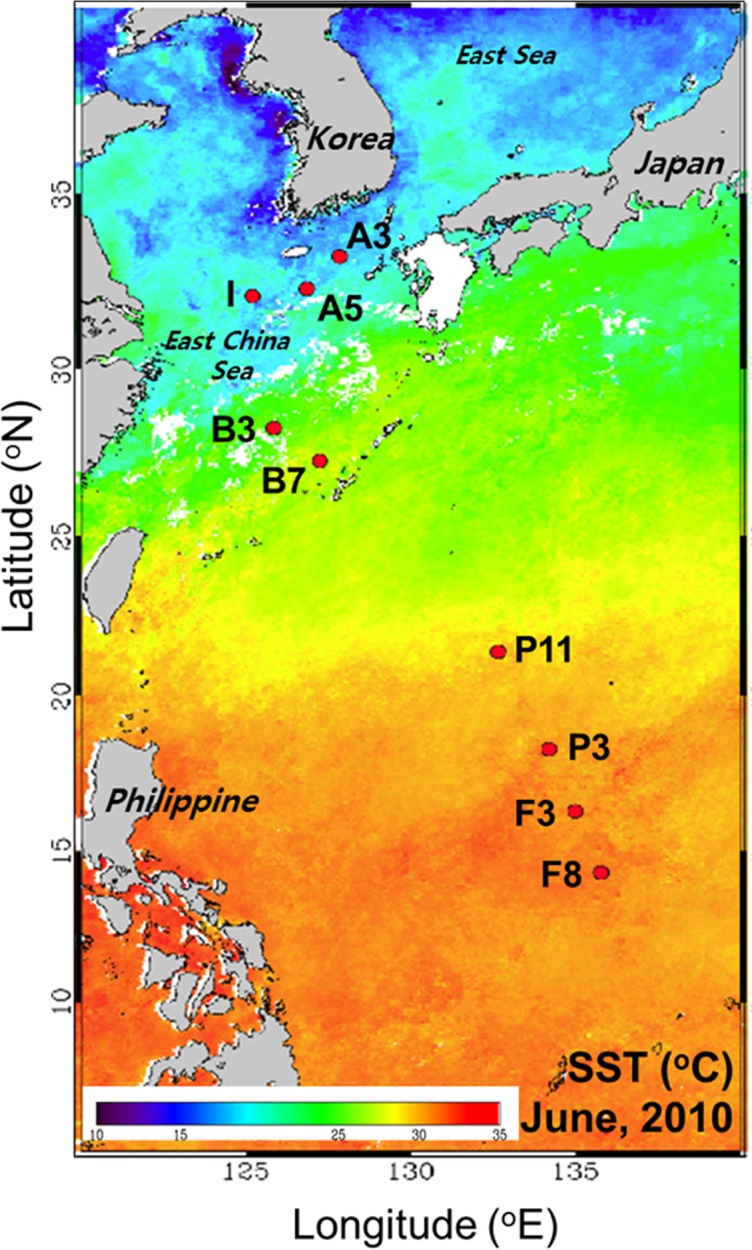
Map of sampling stations in the NW Pacific Ocean. The base map is a composite image of sea surface temperature from 1 to 5 June 2010, obtained from SeaWiFS.

Water samples were collected at nine stations during the NW Pacific Ocean study on the environment and interactions between deep ocean and marginal seas (POSEIDON) cruise in the NW Pacific Ocean from 26 May to 12 June 2010, aboard the R/V Onnuri (Fig. 1). Stations (Stns.) on lines F and P are located in a tropical area affected by the oligotrophic North Equatorial Current (NEC). Stations on line B are located in a subtropical area mainly affected by the oligotrophic Kuroshio Current (KC). Stations A3 and A5 are located in the eastern part of the East China Sea (ECS), through which a branch current of the KC passes. Finally, Stn. I is located in the central area of the ECS and mainly affected by coastal currents [23]. At each station, seawater was sampled at four to six depths between the surface and 150 m using Niskin bottles attached to a rosette sampler. As the sampling sites on the line B are located within the EEZ of Japan, we collected the samples with permission from the Ministry of Foreign Affairs of Japan. For the other stations, no specific permissions were required as samples were taken in domestic or international waters and did not involve endangered or protected species.

### Primers for amplification of *dddP* genes

Amino acid sequences reported to be DddP-like polypeptides were obtained from known bacteria and fungi and from GOS data (refer to S1 Fig. in Todd *et al.* [18]), and used to design primers for *dddP* gene amplification. Using the amino acid sequences, partially degenerate CODEHOP (COnsensus-DEgenerate Hybrid Oligonuceotide Primer) PCR primers were designed by the iCODEHOP program [24]. For pyrosequencing using GS-FLX Titanium, a primer set that was expected to produce an amplicon size of ∼400 bp was selected from the entire set of degenerate primers designed by the program (Table 1). The degenerate core sequences of the forward and reverse primers were made from three (FYF; from 140th amino acid of DddP of *Roseovarius nubinhibens* ISM10994) and four (GEWI; from 264th amino acid) sequences conserved in DddP polypeptides from all known groups, respectively. In addition, the specificity of the designed primers was examined using the PCR–cloning–sequencing approach with selected samples; phylogenetic analysis showed that most clones (62 of 64) were clustered within the *dddP* clade and distributed among all known subclades (G1∼G3).

**Table 1:** Primers used in the pyrosequencing of *dddP* genes.

**Primer**	**Adapter**	**Key**	**MID**	**Specific oligonucleotide** * **(5’ → 3’)**
B1F-fusion (forward)	CCATCTCATCCCTGCGTGTCTCCGAC	TCAG	Variable (10-mers)	**CCGGCGCCGAC**HTNTTYTAYTT
D4R-fusion (reverse)	CCTATCCCCTGTGTGCCTTGGCAGTC	TCAG	None	**CAGCCGGGTCTCG**ATCCAYTCNCC

*Specific oligonucleotides consist of a degenerate ‘core’ (plain text) and non-degenerate ‘clamp’ (bold) region.

### DNA extraction, PCR amplification, and pyrosequencing

Two-liter water samples were passed through a 0.2-μm Supor® filter (47 mm diameter, Gelman Sciences, Ann Arbor, MI, USA), and filters were frozen at −80°C after the addition of 1 ml of STE buffer (100 mM NaCl, 10 mM Tris–HCl, 1 mM EDTA, pH 8.0). For DNA extraction, filters were thawed, cut into small pieces with sterilized scissors, and placed in 50-ml sterile conical tubes. After adding 2 ml of STE buffer, microbial cells were lysed using lysozyme, sodium dodecyl sulfate, and proteinase K according to Somerville et al. [25]. The DNA was then purified from the lysates using a DNeasy Blood and Tissue Kit (Qiagen, Hilden, Germany) following the manufacturer’s instructions.

To amplify *dddP* gene sequences, a two-round PCR method was adapted due to the low efficiency of the fusion primers. We added 1–10 ng of each template DNA to the PCR reaction (total 20 μl), which contained 1× GeneAmp PCR buffer I (Applied Biosystems, Foster City, CA, USA), 0.2 mM of each deoxyribonucleoside triphosphate, 0.5 μM of each primer with only a specific oligonucleotide (Table 1), and 2 units of AmpliTaq Gold DNA polymerase (Applied Biosystems). PCR amplification was conducted according to the following cycle parameters: an initial denaturation step (5 min, 94°C), followed by 30 cycles of denaturation (45 s, 94°C), annealing (45 s, 60°C), and extension (1 min, 72°C), and a final 10-min extension step at 72°C. After gel extraction of the target band using a gel extraction kit (Qiagen), a second round PCR was conducted using the fusion primers and the gel-purified first PCR products as templates (Table 1). Quantification of each final PCR product was performed on agarose gels using DNA QuantLadders (Lonza Rockland Inc., Rockland, ME, USA). Identical quantities of each PCR product were pooled and then purified using the AccuPrep PCR purification kit (Bioneer, Daejeon, Korea). After resolution on 2% agarose gel, the region between 450 and 550 bp was excised and DNA was extracted using a gel extraction kit (Qiagen). Pyrosequencing of PCR products was performed using GS-FLX Titanium (454 Life Sciences, Branford, CT, USA) at Macrogen Co. (Seoul, Korea). Sequence reads from this study were submitted to the National Center for Biotechnology Information (NCBI) sequence read archive (SRA; http://www.ncbi.nlm.nih.gov/Traces/sra; accession number SRX534398).

### Pyrosequencing data analysis

Pyrosequencing data were analyzed using mainly the Qiime (v1.5; [26]) and Mothur softwares [27]. Raw reads were filtered to remove errors by allowing only perfect matches to the barcode and forward primer sequences. The allowed number of maximum homopolymers was 6. All reads <350 bp or >450 bp were removed. The flowgram data were then denoised [28]. Frameshift errors were corrected using the program HMM-FRAME [29]. Among the resulting reads, those without a reverse primer and those <330 bp were removed. In addition, reads were translated into amino acids, and any sequences possessing nonsense mutations were removed. Chimeric sequences were checked using the Perseus program implemented in the Mothur program. Operational taxonomic units (OTUs) were determined for the remaining DNA sequences using the Uclust at a similarity threshold of 90% [30].

### Phylogenetic analysis

Phylogenetic analyses of DddP proteins were conducted using sequences from cultured α-proteobacteria and fungi, GOS metagenomic data [18], and the clone library [21]. In addition, we included genes annotated as *dddP* in the IMG-ER database [31]. After the amino acid sequences were aligned using the muscle program [32], a reference maximum-likelihood tree was constructed using PhyML 3.0 program [33] under JTT substitution model, incorporating a gamma distribution for among-site rate variation and an estimate of invariable sites (JTT + Γ + I). After translation, the phylogenetic positions of each representative sequence for the OTUs were placed onto the reference tree using the pplacer software [34].

### DMS production by *Candidatus* Puniceispirillum marinum IMCC1322

To determine whether a strain belonging to the SAR116 group can produce DMS from DMSP, the IMCC1322 strain, which is one of two isolates belonging to SAR116 [35], was grown in the R2A broth. When the strain grew to an OD_620_ of 0.03, the culture was dispensed in two 30-ml sterile vials. Then, the DMSP substrate (final conc. of 500 μM) was injected into one of the vials, and DMS concentrations in the medium were quantified using DMS extraction, a trapping device, and a gas chromatograph equipped with a flame photometric detector (GF-FPD; [36]). Following the addition of DMSP, 50–100 μl of the samples was removed using a 250-μl gastight syringe (Hamilton 1725RN; Sigma-Aldrich, St. Louis, MO, USA). Sequential DMS measurements were carried out over periods of 15 min to 8 h. Abiotic control, containing the same concentration of DMSP substrate and filtered seawater, was prepared in parallel to correct for any background sources of DMS [37].

Reverse transcription (RT)-PCR was performed to examine expression of the *dddP* gene in IMCC1322 in the absence and presence of the DMSP substrate. Total RNA was extracted using the RNeasy Mini Kit (Qiagen) according to the manufacturer’s instructions. cDNA was prepared from the RNA using the QuantiTech Reverse Transcription Kit (Qiagen). Subsequent PCR was conducted using the primer sets targeting 16S rRNA and *dddP* genes (S1 Table).

### DMS and chlorophyll a concentrations

DMS analysis was conducted by gravitationally filtering 30 ml samples through a 47-mm glass-fiber filter (Whatman GF/F). Filtrates were stored in an amber glass vial with no headspace, and the vial was quickly sealed with a gas-tight cap, the inside of which was coated with Teflon. Within an hour of sampling, 10–20 ml of samples were delivered to the sparging chamber to measure DMS concentration by GC-FPD. The response of GC-FPD was calibrated against both gas standards with certified values of mole fractions of DMS (Scott Specialty Gases^©^, 522 ppbv DMS) and DMS solutions of known concentration prepared by alkaline hydrolysis of DMSP-Cl (Tokyo Kasei Inc.) in an amber vial (30 ml) with a gas tight Teflon cap. Chlorophyll *a* (chl *a*), extracted with 90% acetone, was determined using a Turner fluorometer (10 AU; Turner Designs, Sunnyvale, CA, USA; [38]).

## Results and Discussion

### Characteristics of the pyrosequencing run and OTUs

In this study, two-round of PCR was adapted. The extended PCR cycles might increase PCR artifacts, such as chimera and heteroduplex formations [39], and led to overestimation of species richness [40]. However, each clone library obtained by a normal 30 cycles PCR and two-round PCR with additional 20 cycles were similar (S1 Fig.), indicating that the PCR artifacts might not be significant. Further, we applied strict filtering and screening procedures including chimera removal and correction of frame-shift error,and moreover assigned OTUs at low sequence identity (90%) to minimize PCR artifacts. Thus, the genetic diversity of dddP genes, at least diversity of major groups, may not be seriously affected by the PCR artifacts in this study.

Through strict denoising and screening steps, we obtained a total of 54,548 reads. On the basis of 90% DNA sequence identity, sequences were clustered in 57 OTUs. As the *dddP* gene sequence differences among species belonging to the same genera were generally higher than 10% (S2 Table), the sequences clustered in each OTU likely belonged to the same genus.

Except for one sample (131 sequences of Stn. P3 at a depth of 108 m), the number of sequences among samples varied from 445 to 3,437 (mean ± 1SD, 1158 ± 575). In rarefaction curves, the observed OTU numbers tended to be saturated at each sampling depth (S2 Fig.).

### Phylogenetic diversity of *dddP* genes

**Figure 2:**
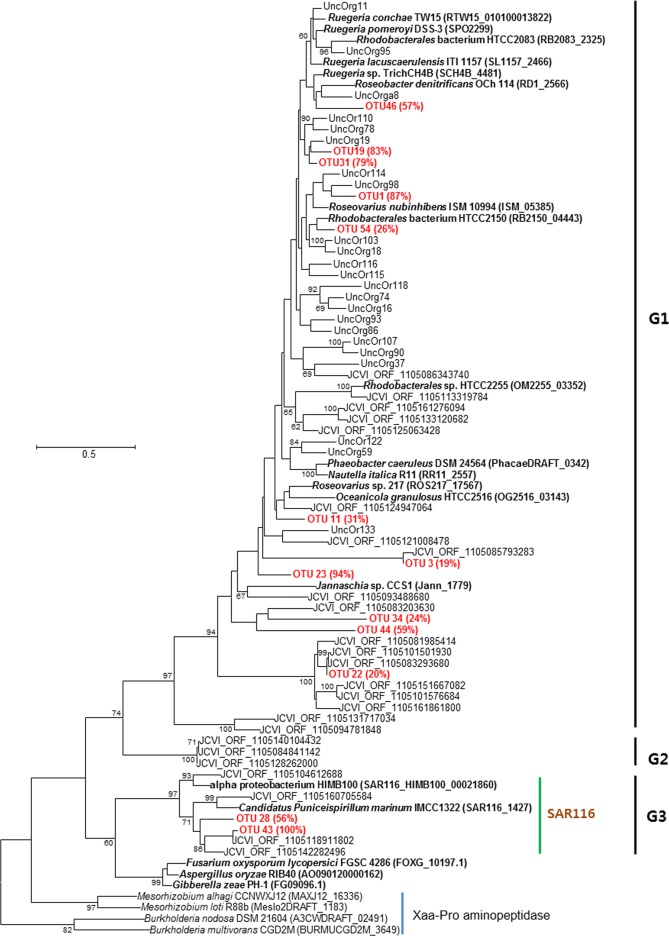
Tree showing the phylogentic relationships of representative amino acid sequences of each OTU obtained in this study (red text). Only OTUs constituting more than 10% of at least one sample are shown. The text in parentheses after bacterial names represent the locus tag for each genome in the IMG/ER database. The percentage in parentheses after OTU names represent maximum percentage of each OTU found in samples. Group names according to Todd *et al*. (2009) are shown on the right. A phylogentic tree for all OTUs can be found in S2 Fig. Only bootstrap values >60% are shown at the nodes.

In the phylogenetic analyses (S3 Fig.), all 57 OTUs were distributed within the putative *dddP* clade, including *dddP* genes of α-proteobacteria and fungi, which are known to produce DMS from DMSP [18]. Among them, 42 were clustered with strains belonging to the Roseobacter clade (G1 group), indicating high diversity of *dddP* genes in the Roseobacter clade. Notably, 11 OTUs were clustered into a clade exclusively composed of two strains belonging to SAR116. DMS production by the SAR116 clade has not been well studied, despite the recognition that a *dddP* homolog exists in the SAR116 genome [13,22]. Although the SAR116 clade belonged to α-proteobacteria, the SAR116 cluster of *dddP* genes formed a sister clade with fungi (Fig. 2), confirming inter-domain horizontal gene transfer from α-proteobacteria to fungi [41].

Among the 57 OTUs, only 13 were dominant and occupied over 10% of at least one sample (Fig. 2). Conversely, 34 OTUs constituted less than 2% of all samples (S3 Fig.), suggesting that they are minor genotypes in the study area.

### Distribution of *dddP* genes in the NW Pacific Ocean

**Figure 3:**
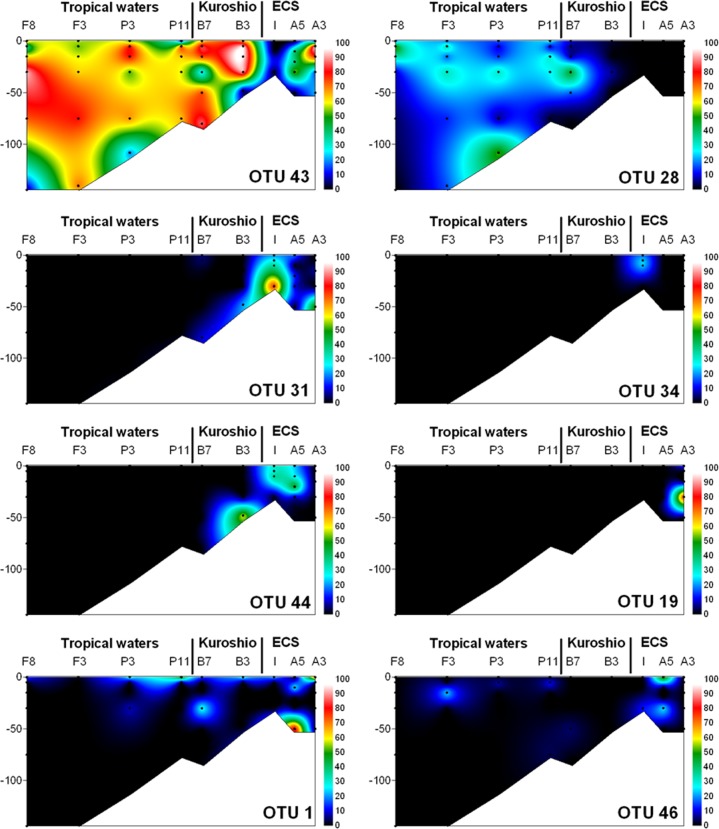
Contour plots showing the percentage of each major OTU as a proportion of all sequences. Black dots represent sampling depths.

Two OTUs affiliated with the SAR116 clade dominated *dddP* genotypes in the euphotic depth of most stations, except Stn I (Fig. 3). Of them, OTU43 was the most dominant genotype in the oligotrophic open ocean and the coastal waters affected by the oligotrophic Kuroshio warm current. OTU28 was also found in the oligotrophic ocean but was less abundant than OTU43, and was not found in coastal waters. In tropical and subtropical waters, the two OTUs showed a complementary relationship, and thus the sum of two *dddP* genes, except the two lower euphotic samples, reached 82 ± 14% (mean ± 1SD). This suggests that bacteria related to SAR116 could play an important functional role in DMS production via DddP lyase.

**Figure 4:**
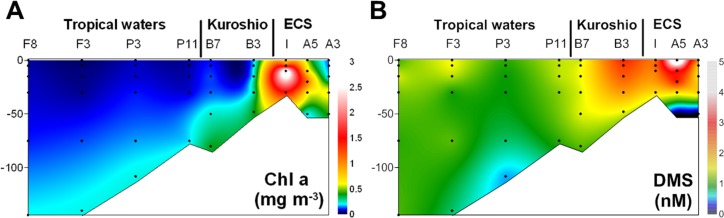
Contour plots showing (A) chl *a* and (B) DMS concentration in the NW Pacific Ocean. Black dots represent the sampling depths.

Despite the fact that during the present study, *dddP* gene sequences in the SAR116 clade were dominant in the surface water of the oligotrophic NW Pacific Ocean with low chl *a* concentrations (Fig. 4A), Todd *et al*. [18] only retrieved five *dddP* gene sequences clustered in the SAR116 clade from the GOS data set. However, in a new BLAST search of the GOS data set using a DddP polypeptide of IMCC1322 as a query and a matching criterion of <e^−80^, 25 homologs clustered into group G3 were additionally retrieved from diverse samples (S4 Fig.). Although the previous study missed these due to the strict criterion for BLAST matching (<e^−80^), the *dddP* sequences in the SAR116 clade seemed to be abundant in the GOS samples.

The composition of DMS-producing bacteria in coastal waters differed from that in oligotrophic open waters (Fig. 3). At Stn. I, having high chl *a* (3.2 μg l^−1^; Fig. 4A) due to influence of coastal water, three OTUs (OTU31, OTU34, and OTU44) clustered into the *Roseobacter* clade comprised of 68%–97% of the total sequences, suggesting that *Roseobacter* clade bacteria could contribute to DMS production via DddP lyase in coastal waters. However, the distribution of *dddP* genotypes in the ECS was largely variable among samples (Fig. 3). As seen in the spatial distribution of picocyanobacteria in the eastern ECS [42], this spatial variation seems to reflect complex physicochemical conditions resulting from the mixing of diverse water masses.

Despite maximal DMS concentration was found at surface of Stn A3 and tended to be higher in the coastal waters than in open waters, the difference of DMS concentrations in euphotic zone between open and coastal waters (mean ± 1SD of 2.4 ± 1.0 nM and 1.1 ± 0.2 nM, respectively) generally varied within 2-folds (Fig. 4B). Considering the vast area of the oligotrophic waters, thus, DMS production seems to be larger in oligotrophic open ocean than in coastal waters. The DddP lyases of SAR116 group were dominant from the oligotrophic tropical waters to the boundary waters of continental shelf. In this respect, the SAR116 group may play a significant role in global DMS production. On the contrary, *Roseobacters* seem to make a more contribution to DMS production in coastal area with relatively high chl *a* and DMS concentrations. Thus, SAR116 and *Roseobacter* groups seem to play a dominant role in DMS production via DddP lyases in the open and coastal waters, respectively.

### DMS production by *Candidatus* Puniceispirillum marinum IMCC1322

**Figure 5:**
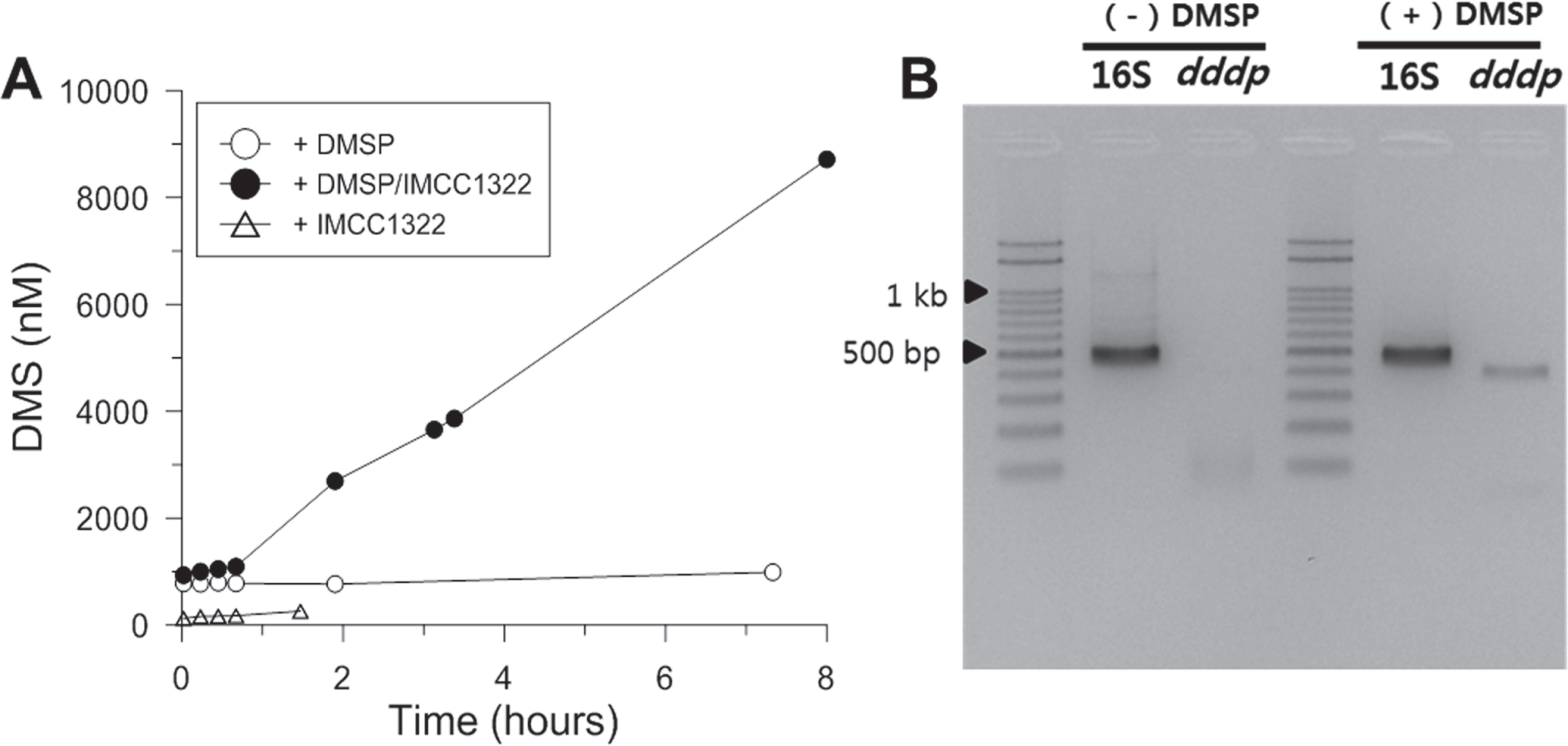
DMS production and expression of *dddP* mRNA of *Candidatus* Puniceispirillum marinum. (A) Closed circles represent cells grown in R2A broth supplemented with 500 μM of DMSP. Open circles and triangles represent controls without cells and without DMSP, respectively. (B) RT-PCR of *dddP* mRNA and 16S rRNA of cells grown in media with DMSP and without DMSP, respectively.

The production of DMS by strains in the SAR116 group has not yet been tested. When a culture of IMCC1322 was enriched with DMSP, DMS production greatly increased (Fig. 5A), confirming that this strain can produce DMS by utilizing DMSP. Furthermore, the transcription level of the *dddP* gene increased in a sample enriched with DMSP (Fig. 5B). As other kinds of putative DMSP lyases are not detected in the IMCC1322 genome yet [35], DMS production in the SAR116 strain most likely occurs via DddP lyase.

In conclusion, although the *Roseobacter* clade was the dominant *dddP*-containing bacteria in coastal areas, the SAR116 group was found to be dominant *dddP*-containing bacteria in the oligotrophic NW Pacific Ocean. Furthermore, we experimentally confirmed DMS production in a SAR116 bacterium, a phenomenon that was neglected in previous studies. In the ocean, the “bacterial switch” between the competing demethylation and cleavage pathways could be regulated by diverse environmental factors such as UV radiation, carbon and sulfur demands, temperature, and DMSP availability [2,43–45]. But regulation mechanisms of the competing pathways have not yet been thoroughly understood. Strains belonging to SAR116 group also have the *dmdA* gene, and therefore participate in the demethylation of DMSP [35]. Furthermore, SAR116 group must be an important member in DMSP cycle in the ocean. Thus, further studies on DMSP metabolism of SAR116 groups would help our understanding of biogeochemical pathways of DMSP and environmental factors regulating these pathways in the ocean.

## Supporting Information

S1 Table Primer sequences used in the RT-PCR.
(DOC)

S2 Table The number of base differences per site between *dddP* gene sequences of the bacterial isolates.
(DOC)

S1 Fig Neighbor-joining tree showing the phylogenetic relationship of clone sequences obtained by a normal 30-cycle PCR (red text) and the two-round PCR (blue text) with additional 20 cycles (see Materials and Methods). The DNA sample obtained at surface of Stn F8 in June, 2013 was used as a template and the primers used in the 1st and 2nd-PCR was shown in the Table 1 and Materials and Methods.
(DOC)

S2 Fig Rarefaction curves of surface sample of each sampling station.
(DOC)

S3 Fig Tree showing the phylogenetic relationships of representative amino acid sequences of each OTU obtained in this study.
(DOC)

S4 Fig Tree showing the phylogenetic positions of sequences (bold text) retrieved in this study from the GOS database.
(DOC)
