# Supplementary material for: Pyrosequencing Revealed SAR116 Clade as Dominant dddP-Containing Bacteria in Oligotrophic NW Pacific Ocean
Source: PLoS One. 2015 Jan 23;10(1):e0116271. doi: 10.1371/journal.pone.0116271 (PMC4304780; doi:10.1371/journal.pone.0116271)
Supplement: S1 Table — Primer sequences used in the RT-PCR. (DOC) [file pone.0116271.s001.doc]

**Table S1**. Primer sequences used in the RT-PCR. The target DNA was amplified in a 50-μl reaction containing 1 U of Ex-Taq (TaKaRa, Kyoto, Japan), 0.2 μM of each primer, 0.2 mM of each dNTP, 1× PCR buffer, and 1 μl cDNA. PCR was conducted according to the following parameters: an initial denaturation step (5 min, 94°C) followed by 30 cycles of denaturation (45 s, 94°C), annealing (45 s, 60°C), and extension (1 min, 72°C), and a final 10-min extension step at 72°C.

| Target gene | Name | Sequences (5' -> 3') | Amplicon size  (bps) |
| --- | --- | --- | --- |
| dddP | DddP_1322-F | GAAGGTAAGATGATTCTG | 414 |
|  | DddP_1322-R | TAATGTTGCCCATAATTC |  |
| 16S rRNA | 16S-F | AAAGATTTATCGGTGATA | 476 |
|  | 16S-R | CAATATCTACGAATTTCA |  |
