# Supplementary material for: Pyrosequencing Revealed SAR116 Clade as Dominant dddP-Containing Bacteria in Oligotrophic NW Pacific Ocean
Source: PLoS One. 2015 Jan 23;10(1):e0116271. doi: 10.1371/journal.pone.0116271 (PMC4304780; doi:10.1371/journal.pone.0116271)
Supplement: S2 Table — The number of base differences per site between dddP gene sequences of the bacterial isolates. (DOC) [file pone.0116271.s002.doc]

**Table S2**. The number of base differences per site between *dddP* gene sequences of the bacterial isolates.
