# Supplementary material for: Pyrosequencing Revealed SAR116 Clade as Dominant dddP-Containing Bacteria in Oligotrophic NW Pacific Ocean
Source: PLoS One. 2015 Jan 23;10(1):e0116271. doi: 10.1371/journal.pone.0116271 (PMC4304780; doi:10.1371/journal.pone.0116271)
Supplement: S1 Fig — The DNA sample obtained at surface of Stn F8 in June, 2013 was used as a template and the primers used in the 1st and 2nd-PCR was shown in the Table 1 and Materials and Methods. (DOC) [file pone.0116271.s003.doc]

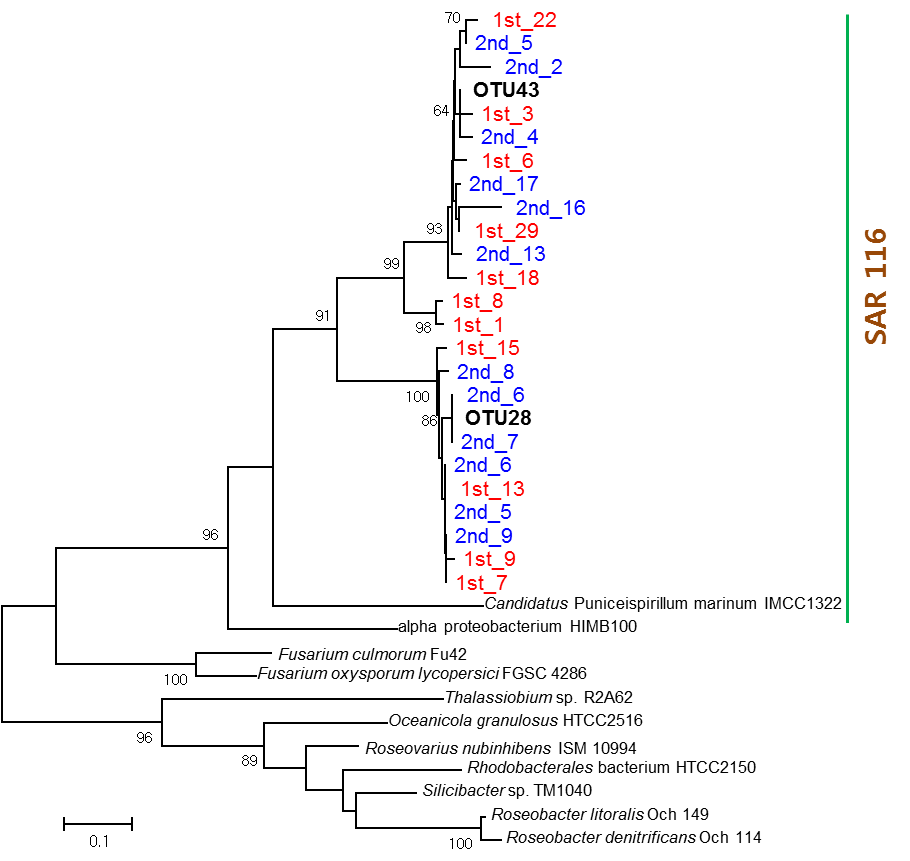


**Figure S1**. Neighbor-joining tree showing the phylogenetic relationship of clone sequences obtained by a normal 30-cycle PCR (red text) and the two-round PCR (blue text) with additional 20 cycles (see Materials and Methods). The DNA sample obtained at surface of Stn F8 in June, 2013 was used as a template and the primers used in the 1st and 2nd-PCR was shown in the Table 1 and Materials and Methods.
