# Supplementary figures and images for: Pyrosequencing Revealed SAR116 Clade as Dominant dddP-Containing Bacteria in Oligotrophic NW Pacific Ocean
Source: PLoS One. 2015 Jan 23;10(1):e0116271. doi: 10.1371/journal.pone.0116271 (PMC4304780; doi:10.1371/journal.pone.0116271)

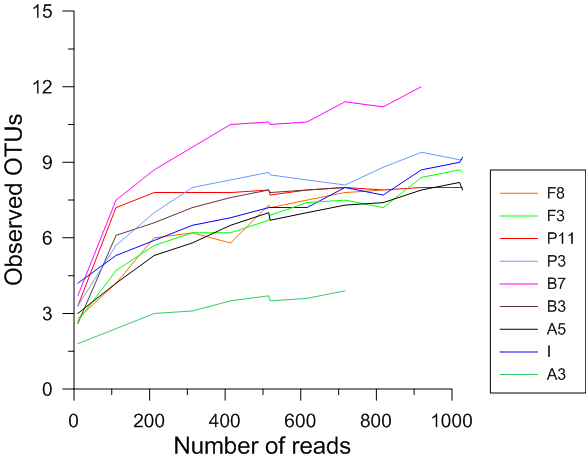


**Figure S2**. Rarefaction curves of surface sample of each sampling station

Supplement: S2 Fig — Rarefaction curves of surface sample of each sampling station. (DOC) [file pone.0116271.s004.doc]
