# Supplementary material for: Pyrosequencing Revealed SAR116 Clade as Dominant dddP-Containing Bacteria in Oligotrophic NW Pacific Ocean
Source: PLoS One. 2015 Jan 23;10(1):e0116271. doi: 10.1371/journal.pone.0116271 (PMC4304780; doi:10.1371/journal.pone.0116271)
Supplement: S3 Fig — Tree showing the phylogenetic relationships of representative amino acid sequences of each OTU obtained in this study. (DOC) [file pone.0116271.s005.doc]

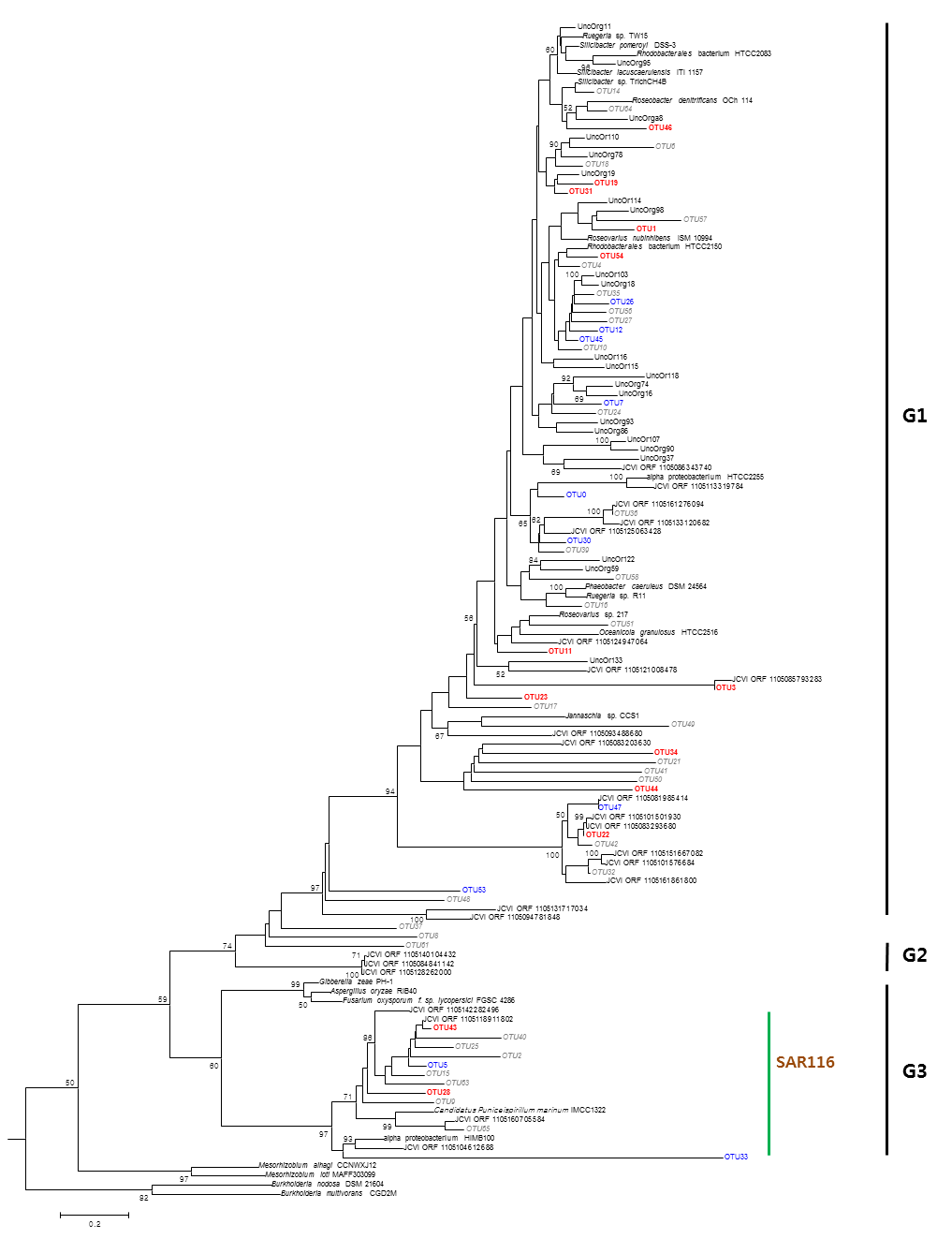


**Figure S3**. Tree showing the phylogenetic relationships of representative amino acid sequences of each OTU obtained in this study. Red, blue, and gray text colors represent OTUs constituting >10% and >2% of at least one sample and <2% of all samples, respectively.
