# Supplementary material for: Pyrosequencing Revealed SAR116 Clade as Dominant dddP-Containing Bacteria in Oligotrophic NW Pacific Ocean
Source: PLoS One. 2015 Jan 23;10(1):e0116271. doi: 10.1371/journal.pone.0116271 (PMC4304780; doi:10.1371/journal.pone.0116271)
Supplement: S4 Fig — Tree showing the phylogenetic positions of sequences (bold text) retrieved in this study from the GOS database. (DOC) [file pone.0116271.s006.doc]

**Figure S4**. Tree showing the phylogenetic positions of sequences (bold text) retrieved in this study from the GOS database.
